# Multi-Chaotic HEOA for Hardware-Aware Neural Architecture Search: Brain Tumor Classification on FPGA

**DOI:** 10.3390/s26092822

**Published:** 2026-05-01

**Authors:** Ismail Mchichou, Hamza Tahiri, Mohamed Amine Tahiri, Hicham Amakdouf

**Affiliations:** 1 Laboratory of Electronic Signals and Systems of Information, Dhar El Mahrez Faculty of Science, Sidi Mohamed Ben Abdellah University, Fez 30000, Morocco; ismail.mchichou@usmba.ac.ma (I.M.);; 2 Engineering, Systems and Applications Laboratory, National School of Applied Sciences, Sidi Mohamed Ben Abdellah University, Fez 30000, Morocco

**Keywords:** neural architecture search, multi-chaotic optimization, HEOA, brain tumor classification, FPGA implementation, deep learning, medical image analysis, embedded systems, high-level synthesis, Zynq-7000

## Abstract

Automated brain tumor classification from MRI scans requires optimized CNN architectures deployable on embedded FPGA platforms. This paper presents an integrated approach combining the Multi-Chaotic Enhanced HEOA (MC-HEOA) for automatic CNN architecture discovery with deployment validation on a Xilinx Zynq-7000 FPGA. A CEC2023 benchmark across 10 test functions evaluates 6 chaotic maps and selects the Tent map as the optimal diversity generator. The NAS search space spans a massive combinatorial space of $1.31\;\times \;10^{16}$ configurations encoding architectural choices (layers, convolutions, channels, pooling) under a strict constraint of fewer than one million parameters for FPGA compatibility. The optimal discovered architecture, trained and evaluated using single-channel grayscale input (224 × 224 × 1)—the natural representation for intrinsically monochromatic MRI data— achieves 91.33% test accuracy and 92.44% validation accuracy with 724,200 parameters on the 4-class Brain Tumor MRI dataset (glioma, meningioma, pituitary, no tumor). HLS synthesis on the Zynq-7000 (xc7z020clg484-1) validates embedded deployment feasibility, with DSP utilization of 16%, LUT utilization of 57%, FF utilization of 28%, and an inference latency of 374 ms at 100 MHz. This study demonstrates the effectiveness of MC-HEOA for discovering compact, high-performing CNN architectures compatible with FPGA deployment, opening new perspectives for real-time embedded medical diagnosis.

## 1. Introduction

Convolutional neural networks (CNNs) have demonstrated remarkable performance across a wide range of medical computer vision tasks, including pathology classification, lesion detection, and organ segmentation [1,2,3]. Large-scale benchmark challenges such as MoNuSAC2020 [4] have further demonstrated that deep CNN architectures can achieve performance comparable to inter-human agreement on complex histological analysis tasks while highlighting the critical importance of architectural choices and optimization strategies for medical imaging applications. Automated brain tumor classification from magnetic resonance imaging (MRI) represents a major clinical challenge [5,6,7,8,9,10], requiring reliable, fast diagnostic assistance systems deployable in resource-constrained medical environments. Recent Deep Learning approaches for brain tumor classification [1,2,3] have demonstrated promising performance, achieving accuracies of 95–99% on public datasets [5,6,9,10] yet at the cost of large architectures (25–138 M parameters) that are ill-suited for embedded deployment. The deployment of CNNs on resource-constrained embedded systems remains a considerable technical challenge due to their substantial computational and memory requirements. Field-Programmable Gate Array (FPGA) platforms represent an attractive solution for embedded neural network inference [11,12,13], offering a compelling trade-off among performance, reconfiguration flexibility, and energy efficiency compared to general-purpose processors and GPUs. Recent implementations have demonstrated the effectiveness of FPGAs for medical imaging applications [14,15], real-time classification [16,17,18,19], and adaptive optimization [20,21], with significant gains in latency and energy consumption. Nevertheless, designing FPGA-optimized CNN architectures demands deep hardware design expertise and considerable development time, thereby limiting their adoption in embedded medical applications.

Neural Architecture Search (NAS) has emerged as a promising solution for automating this design process [22,23,24]. Existing NAS approaches have demonstrated their ability to discover high-performing architectures for diverse tasks, sometimes surpassing manually crafted expert designs [25,26,27]. Recent advances include training-free evolutionary optimization [26] and the use of attention mechanisms for path evaluation [27]. However, as highlighted in several recent surveys [22,23,24], the majority of current NAS methods primarily optimize model accuracy while relying on generic metrics such as parameter count or floating-point operations (FLOPs) as proxies for computational efficiency. These metrics do not faithfully reflect the actual hardware constraints of FPGA platforms, particularly the limited availability of logic resources (LUTs), embedded memory (BRAM), and dedicated compute units (DSPs). Although recent hardware-aware NAS approaches [28,29,30,31] have been proposed to integrate hardware constraints during the search, these methods primarily target dynamic architectures [28,31] or graph networks [29], with limited focus on the a priori integration of strict FPGA constraints for embedded medical applications. As a result, architectures discovered by conventional methods frequently require a post-search compression or quantization phase to satisfy hardware constraints, significantly degrading their initial performance or rendering deployment impractical on System-on-Chip FPGA devices such as the Xilinx Zynq-7000.

Metaheuristic evolutionary optimization algorithms are widely employed across various complex optimization contexts [32,33,34], with notable successes for Particle Swarm Optimization (PSO), Genetic Algorithms (GAs), Differential Evolution (DE) [35,36,37], and more recently the Human Evolutionary Optimization Algorithm (HEOA) [38]. Recent comparative studies [32,33,35,37] have shown that the performance of these algorithms is strongly problem-dependent and sensitive to the adopted optimization strategy, including cooperative [36] and AI-hybrid [39] approaches. Our prior work [20] explored real-time contextual optimization on FPGA through dynamic chaotic map selection and adaptive metaheuristics, demonstrating the potential of chaotic approaches to enhance search diversity. HEOA draws inspiration from human evolutionary and social learning mechanisms to guide the search toward promising regions of the solution space. On certain optimization problems, HEOA can achieve rapid convergence through its adaptive learning strategies. However, when applied to complex optimization landscapes with multiple constraints—particularly in the context of NAS under hardware constraints—the original HEOA exhibits several limitations: insufficient search diversity stemming from its exclusive reliance on the logistic map for chaotic generation, sensitivity to static control parameters, and susceptibility to premature convergence on highly multi-modal problems.

To address these limitations, this work proposes an integrated approach combining HEOA enhancement through empirical selection of the optimal chaotic map with CNN architecture discovery under strict FPGA constraints.

The main contributions of this work are:

1. An MC-HEOA framework that enhances the original HEOA through empirical selection of the Tent chaotic map via CEC2023 benchmarking, strengthening the exploration–exploitation balance.
2. The a priori integration of Zynq-7000 FPGA constraints (<1 M parameters, BRAM compatibility) into the NAS objective function, ensuring directly deployable architectures without post-search compression.
3. The automatic discovery of a CNN architecture achieving 91.33% test accuracy with 724,200 parameters on the Brain Tumor MRI dataset (4 classes: glioma, meningioma, pituitary, no tumor).
4. Zynq-7020 FPGA validation confirming embedded deployment feasibility with an inference latency of 374 ms at 100 MHz and acceptable resource utilization.

The remainder of this paper is organized as follows. Section 2 details the theoretical foundations of the original HEOA and introduces the six candidate chaotic maps. Section 3 describes the proposed methodology, including the MC-HEOA framework, the NAS search space, FPGA constraint integration, and the training and hardware implementation protocol. Section 4 presents and analyzes experimental results on the CEC2023 benchmark, NAS-based architecture discovery, classification performance, and FPGA validation. Section 5 discusses the interpretation of results, comparison with the state of the art, study limitations, and practical implications. Finally, Section 6 concludes this paper and outlines directions for future research.

## 2. Human Evolutionary Optimization Algorithm

This section presents the theoretical foundations of the original HEOA, which serves as the conceptual basis for our proposed multi-chaotic extension.

### 2.1. Inspiration and Modeling of HEOA

The Human Evolutionary Optimization Algorithm (HEOA) [38] is a novel metaheuristic inspired by the processes of human evolution and social learning. Its primary inspiration stems from the remarkable adaptive capacity of human evolution and its ability to discover optimal solutions in complex environments. The human evolutionary process, a fundamental driver of survival and societal progress, highlights the effectiveness of natural selection and adaptation.

Unlike traditional evolutionary algorithms that model animal behaviors (bee swarms, ant colonies) or physical phenomena (gravitation, electromagnetism), HEOA specifically simulates the fundamental cognitive mechanisms that enabled humanity to thrive in complex and changing environments. This biomimetic approach provides a particularly effective adaptive capacity for optimization problems requiring a dynamic balance between exploitation and exploration.

HEOA divides the global search process into two distinct phases reflecting human societal evolution: human exploration and human development. Although this division simplifies the complex nature of human evolution, it provides a solid scientific basis for understanding the progression of human societies.

### 2.2. Initialization via Logistic Chaotic Map

To simulate the chaotic nature of the early stages of human evolution, HEOA initializes the population using the logistic chaotic map. This framework offers a distinctive perspective on the origin and evolution of the universe, positing that the universe progressively evolves through chaotic processes and self-organization phenomena.

The initialization formula for a population of size *N* is expressed as:

(1) $$x_{i}=\alpha \cdot x_{i-1}\cdot \left(1-x_{i-1}\right),\;0\leq x_{0}\leq 1,\;i=1,2,\ldots ,N,\;\alpha =4$$

The chaotic value $x_{i}$ is then mapped onto the search space:

(2) $$X_{i}^{0}=\mathrm{lb}+\left(\mathrm{ub}-\mathrm{lb}\right)\cdot x_{i}$$

where lb and ub denote the lower and upper bounds of the search space, respectively.

### 2.3. Human Exploration Phase

During the human exploration phase (defined as the first quarter of the maximum iterations), individuals face novel environments with limited knowledge, adopting a uniform search strategy. This phase incorporates several sophisticated mechanisms inspired by primitive human evolution.

The update equation during this phase is expressed as:

(3) $$\begin{array}{cc} X_{i}^{t+1} & =\beta \left(1-\frac{t}{\mathrm{Maxiter}}\right)\left(X_{i}^{t}-X_{\mathrm{best}}\right)\cdot \mathrm{Levy}\left(\dim\right)\\ \; & \;+X_{\mathrm{best}}\left(1-\frac{t}{\mathrm{Maxiter}}\right)+\left(X_{\mathrm{mean}}^{t}-X_{\mathrm{best}}\right)\cdot \mathrm{floor}\left(\frac{\mathrm{rand}}{f_{\mathrm{jump}}}\right)\cdot f_{\mathrm{jump}} \end{array}$$

The associated parameters and functions are defined as follows:

**Mean position**  $X_{\mathrm{mean}}^{t}$: The average position of the current population, computed as:

(4) $$X_{\mathrm{mean}}^{t}=\frac{1}{N}\sum_{k=1}^{N}X_{k}^{t}$$

**Adaptive function**  β: Responsible for parameter adjustment based on the iteration count and current position. This function accounts for the increasing difficulty of human knowledge exploration and swarming characteristics:

(5) $$\beta =0.2\left(1-\frac{t}{\mathrm{Maxiter}}\right)\cdot \left(X_{i}^{t}-X_{\mathrm{mean}}^{t}\right)$$

**Lévy distribution**: To simulate the complex nature of knowledge acquisition during the human exploration phase and its spiral development characteristic, the Lévy distribution expression is given in Equation (6), where γ is assigned a value of 1.5:

(6) $$\left\{\begin{array}{c} \mathrm{Levy}\left(D\right)=\frac{\mu \cdot \sigma }{{\left|v\right|}^{1/\gamma }}\\ \mu ∼N(0,D)\\ v∼N(0,D)\\ \sigma ={\left[\frac{\Gamma (1+\gamma )\sin(\pi \gamma /2)}{\Gamma \left(\left(1+\gamma \right)/2\right)\;\gamma \;2^{(\gamma -1)/2}}\right]}^{1/\gamma } \end{array}\right.$$

**Jump strategy**: The human exploration phase incorporates a jump strategy inspired by image reframing and reorganization techniques, aimed at improving the dispersion of search locations. This technique leverages the fact that the human eye perceives an image through a combination of global and local features, making it relatively insensitive to the loss of specific local information. The jump coefficient $f_{\mathrm{jump}}$ quantifies the extent of the jump and is expressed as:

(7) $$f_{\mathrm{jump}}=\frac{\mathrm{lb}(1)-\mathrm{ub}(1)}{\delta },\;\delta \in \left[100,\;2000\right]$$

### 2.4. Human Development Phase

In the human development phase, HEOA categorizes the human society into four distinct roles based on individual performance, each employing specialized search strategies. Each role adopts a unique search strategy, and collectively they collaborate to explore the global optimal solution:

**Leaders (top 40%)**: Possessing a wealth of knowledge, they are typically located in optimal regions. Individuals in the top 40% of pre-fitness are designated as leaders. They seek out superior regions for human development by drawing on their existing knowledge. This exploration process is captured by:

(8) $$X_{i}^{t+1}=\left\{\begin{array}{cc} \omega \cdot X_{i}^{t}\cdot \exp\left(-\frac{t}{\mathrm{rand}\cdot \mathrm{Maxiter}}\right), & \mathrm{if}\;R<A\\ \omega \cdot X_{i}^{t}+R_{n}\cdot \mathrm{ones}\left(1,\dim\right),\; & \mathrm{if}\;R\geq A \end{array}\right.$$

In Equation (8), $R_{n}$ is a normally distributed random number. The function ones(1, dim) generates a row vector of dim elements all equal to 1. *R* is a random number in [0, 1] representing the situational complexity associated with leaders. *A* denotes the situation assessment value, set to 0.6 in the conducted experiments. Based on the complexity of the situation at a given position, the leader selects an appropriate search strategy. The knowledge acquisition ease coefficient ω decreases progressively as development advances:

(9) $$\omega =0.2\cos\left(\frac{\pi }{2}\left(1-\frac{t}{\mathrm{Maxiter}}\right)\right)$$

**Explorers (40–80%)**: They play a crucial role by venturing into unexplored territory in search of the global optimal solution. Individuals ranked between the top 40% and 80% of the population by fitness are designated as explorers. Their search strategy is represented by:

(10) $$X_{i}^{t+1}=R_{n}\cdot \exp\left(\frac{{\left(X_{\mathrm{worst}}^{t}\right)}^{2}-{\left(X_{i}^{t}\right)}^{2}}{2}\right)$$

In Equation (10), $X_{\mathrm{worst}}^{t}$ denotes the position of the least fit individual in the population at iteration *t*.

**Followers (80–90%)**: They adhere to the guidance of the most adaptable leader and follow in their footsteps. Specifically, individuals ranked between the top 80% and 90% of the population by fitness level are assigned the follower role. Their search strategy is expressed as:

(11) $$X_{i}^{t+1}=X_{i}^{t}+\omega \cdot R_{d}\cdot \left(X_{\mathrm{best}}^{t}-X_{i}^{t}\right)$$

In Equation (11), $X_{\mathrm{best}}^{t}$ denotes the position of the fittest individual in the population at iteration *t*. $R_{d}$ is a random number in [1,dim].

**Losers (90–100%)**: Individuals that are poorly adapted and remain in the population are termed losers. These under-adapted individuals who fail to integrate into society are eliminated, and the population is replenished through reproduction in regions suitable for human development. The replenishment equation is:

(12) $$X_{i}^{t+1}=X_{\mathrm{best}}+\left(X_{\mathrm{best}}-X_{i}^{t}\right)\cdot R_{n}$$

### 2.5. Computational Complexity of HEOA

The computational complexity of HEOA is governed by three operations: solution initialization, fitness function evaluation, and solution update. Denoting the number of solutions as *N*, the initialization complexity is O(N). The solution update complexity is O(T×N)+O(T×N×dim)+O(T×N×logN), encompassing best-position identification and update of all solution positions, where *T* denotes the total number of iterations and dim the problem dimensionality.

Consequently, the total computational complexity of HEOA is:

O(T×N)+O(T×N×dim)+O(T×N×logN)

### 2.6. Critical Analysis of Original HEOA Limitations

Despite demonstrating promising performance on various optimization functions, the original HEOA exhibits several critical limitations that directly motivate our multi-chaotic extension:

**Restricted chaotic diversity**: Exclusive reliance on the logistic map considerably limits the algorithm’s adaptability to different types of optimization landscapes. This single-map chaotic approach fails to fully exploit the diversified potential of chaotic systems for adaptive exploration and exploitation.

**Parameter rigidity**: The control coefficients (α, β, γ) remain static throughout the entire optimization process, preventing adaptation to the evolving characteristics of the fitness landscape. This rigidity limits algorithmic efficiency when problem properties vary across regions of the search space.

**Absence of adaptive mechanisms**: The original HEOA lacks any feedback system for automatically adjusting its strategies based on detected problem characteristics, the current search evolution phase, or the dynamic properties of the fitness landscape.

**Premature convergence**: The algorithm may stagnate in local optima on highly multi-modal or high-dimensional functions—particularly those of the CEC-2023 standard—limiting its effectiveness on contemporary optimization problems.

These fundamental limitations motivate the integration of six chaotic maps with empirical selection and their application to neural architecture search under FPGA constraints in our multi-chaotic HEOA approach.

### 2.7. Chaotic Maps for Exploratory Diversity

Chaotic systems exhibit fundamental properties that make them particularly well suited for metaheuristic optimization algorithms: sensitivity to initial conditions, ergodicity, and uniform distribution in phase space. These properties enable more diversified exploration of the search space compared to classical pseudo-random number generators. Sensitivity to initial conditions is quantified by the Lyapunov exponent λ > 0, where higher values correspond to faster divergence of trajectories.

Six chaotic maps exhibiting complementary dynamical behaviors are described mathematically below.

**Logistic Map:**

(13) $$x_{n+1}=4x_{n}\left(1-x_{n}\right),\;x_{n}\in \left[0,\;1\right],\;\lambda \approx 0.693$$

**Tent Map:**

(14) $$x_{n+1}=\left\{\begin{array}{cc} \frac{x_{n}}{0.7} & \mathrm{if}\;x_{n}<0.7\\ \frac{1-x_{n}}{0.3} & \mathrm{if}\;x_{n}\geq 0.7 \end{array}\right.,\;\lambda \approx 0.611$$

**Sine Map:**

(15) $$x_{n+1}=\sin\left(\pi x_{n}\right),\;x_{n}\in \left[0,\;1\right],\;\lambda \approx 0.452$$

**Hénon Map (2D):**

(16) $$\left\{\begin{array}{c} x_{n+1}=1-1.4x_{n}^{2}+y_{n}\\ y_{n+1}=0.3x_{n} \end{array}\right.,\;\lambda \approx 0.419$$

**Chebyshev Map:**

(17) $$x_{n+1}=\cos\left(4\arccos\left(x_{n}\right)\right),\;x_{n}\in \left[-1,1\right],\;\lambda \approx 1.386$$

**Circle Map:**

(18) $$x_{n+1}=x_{n}+0.5-\frac{1.5}{2\pi }\sin\left(2\pi x_{n}\right)\;\left(\mathrm{mod}\;1\right),\;\lambda \approx 0.392$$

These maps are integrated into HEOA by substituting pseudo-random numbers in the population update equations (Equations (3) and (8)). An empirical comparative evaluation of these six chaotic maps identifies the optimal generator, which is subsequently integrated into MC-HEOA for neural architecture search. The results of this evaluation are presented in Section 4.

## 3. Proposed Methodology

This section presents the methodology developed for the automatic discovery of convolutional neural network architectures optimized for brain tumor classification from MRI, with implementation validation on a Xilinx Zynq-7000 FPGA. The approach consists of two main phases forming a complete algorithm-hardware co-design chain: HEOA enhancement through chaotic integration and empirical selection of the optimal map (Phase 1), followed by neural architecture search under FPGA constraints with training and hardware validation (Phase 2). This methodology ensures that the optimized architecture is directly deployable on the target hardware without major post-search modifications.

### 3.1. Overview of the Approach

**Figure 1:**
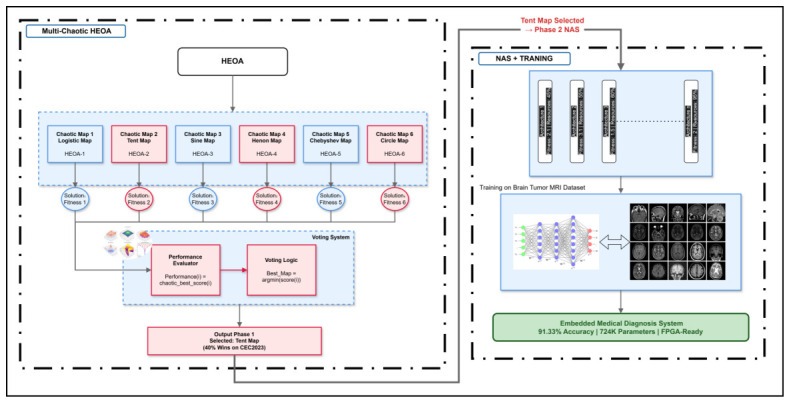
Overview of the MC-HEOA methodology for neural architecture search under FPGA constraints.

The proposed methodology is structured around two complementary phases illustrated in Figure 1.

The first phase enhances the original HEOA by integrating an optimal chaotic map for exploratory diversity generation. The MC-HEOA (Multi-Chaotic Enhanced HEOA) framework designates the methodological approach of empirically evaluating several candidate chaotic maps on standard optimization functions, objectively selecting the map offering the best performance, and subsequently integrating this selected map into the HEOA mechanisms for the NAS application. The comparative evaluation results identified the Tent map as the optimal chaotic generator among six candidates (Logistic, Tent, Sine, Hénon, Chebyshev, Circle). This approach ensures a rigorous, empirically validated selection of the chaotic generator rather than an arbitrary choice.

The second phase leverages the Tent-enhanced HEOA for neural architecture search under strict FPGA constraints, training of the discovered architecture on the Brain Tumor MRI dataset, and hardware implementation validation on the Zynq-7020 FPGA via HLS synthesis. This integrated approach constitutes a distinctive methodological contribution: embedding FPGA constraints directly within the NAS phase guarantees that discovered architectures are intrinsically deployable on embedded hardware without requiring major post-search modifications, while FPGA validation experimentally confirms deployment feasibility.

### 3.2. Phase 1: HEOA Enhancement via Chaotic Integration

The first phase enhances the original HEOA by integrating an optimal chaotic map for exploratory diversity generation. The original HEOA relies exclusively on the logistic map for population initialization, limiting its adaptability to different types of optimization landscapes. Chaotic systems exhibit distinct ergodic properties characterized by their Lyapunov exponent, phase space probability distribution, and temporal autocorrelation, which directly influence exploration quality.

The MC-HEOA framework designates the methodological approach of empirically evaluating several candidate chaotic maps, objectively selecting the optimal map according to performance and robustness criteria, and then systematically integrating this map into all diversity-generating HEOA mechanisms. A comparative evaluation of six chaotic maps (Logistic, Tent, Sine, Hénon, Chebyshev, Circle) on the CEC2023 benchmark (10 functions, 30 runs per function) identified the Tent map as the optimal generator, owing to its perfect uniform distribution over the unit interval and its moderate Lyapunov exponent promoting balanced exploration.

Tent map integration is performed in three key HEOA components. First, population initialization replaces logistic-map generation with Tent-map generation (Equation (14)), applying the transformation iteratively from a random initial condition. Second, during the exploration phase, the random components in Equation (3) are replaced by Tent chaotic sequences, ensuring uniform exploration during the first quarter of iterations. Third, an adaptive reinitialization mechanism replaces the 20% least-performing individuals with new solutions generated via the Tent map whenever the population standard deviation falls below the critical threshold of 0.01, preserving global exploration while retaining high-quality solutions. In the implementation presented, only the Tent map is used during neural architecture search (Phase 2); the five remaining maps served exclusively for the empirical selection phase.

### 3.3. Phase 2: Architecture Search, Training, and FPGA Validation

The second phase integrates three components forming a complete co-design cycle: automatic architecture search via Tent-HEOA under strict FPGA constraints, training of the discovered architecture on the Brain Tumor MRI dataset, and hardware implementation validation on the Zynq-7020 FPGA (Xilinx, San Jose, CA, USA). This integrated approach ensures that the optimized architecture is directly deployable on the embedded platform without major post-search modifications.

#### 3.3.1. Neural Architecture Search Under FPGA Constraints

The NAS problem is formulated as a constrained multi-objective optimization problem aimed at maximizing classification accuracy while minimizing architectural complexity, subject to strict hardware resource constraints:

(19) $$\begin{array}{cc} \max_{x\in X}\; & f_{1}\left(x\right)={\mathrm{Accuracy}}_{\mathrm{val}}\left(x\right)\\ \min_{x\in X}\; & f_{2}\left(x\right)=\mathrm{Params}\left(x\right)\\ s.t.\; & \mathrm{Params}\left(x\right)<10^{6}\\ \; & \mathrm{Layers}(x)\in [2,\;10]\\ \; & x\in {\left[0,\;1\right]}^{28} \end{array}$$

where X denotes the encoded search space, ${\mathrm{Accuracy}}_{\mathrm{val}}$ the validation accuracy on the Brain Tumor MRI dataset, and Params the total number of architecture parameters.

The constraint $\mathrm{Params}\left(x\right)<10^{6}$ constitutes the central element of the proposed co-design approach, derived directly from the hardware specifications of the target Zynq-7020 FPGA. This limit is not arbitrary but follows from a rigorous analysis of available memory resources. The Zynq-7020 (xc7z020clg484-1) provides 280 BRAM blocks of 36 Kbits each, yielding a total capacity of 10,080 Kbits (1.26 MB). In standard float32 representation, each neural network parameter requires 4 bytes of storage. Consequently, one million parameters require 4 MB of memory, approximately three times the total BRAM capacity.

During inference, BRAM must simultaneously accommodate network weights, intermediate feature maps, and computation buffers for convolutional operations. As confirmed by the HLS synthesis results presented in Section 4.6, the discovered architecture (724,200 parameters) utilizes 80% of available BRAM (224/280 blocks), with network weights streamed from the 512 MB DDR3 external memory via AXI interfaces. The $10^{6}$-parameter constraint ensures that the complete inference pipeline remains feasible within the available hardware resources, leaving a 20% safety margin for synthesis variations. This differs from conventional NAS methods that optimize accuracy first and adapt to hardware limitations afterward—an approach that frequently proves unsuccessful for embedded FPGA devices such as the Zynq-7000.

**Figure 2:**
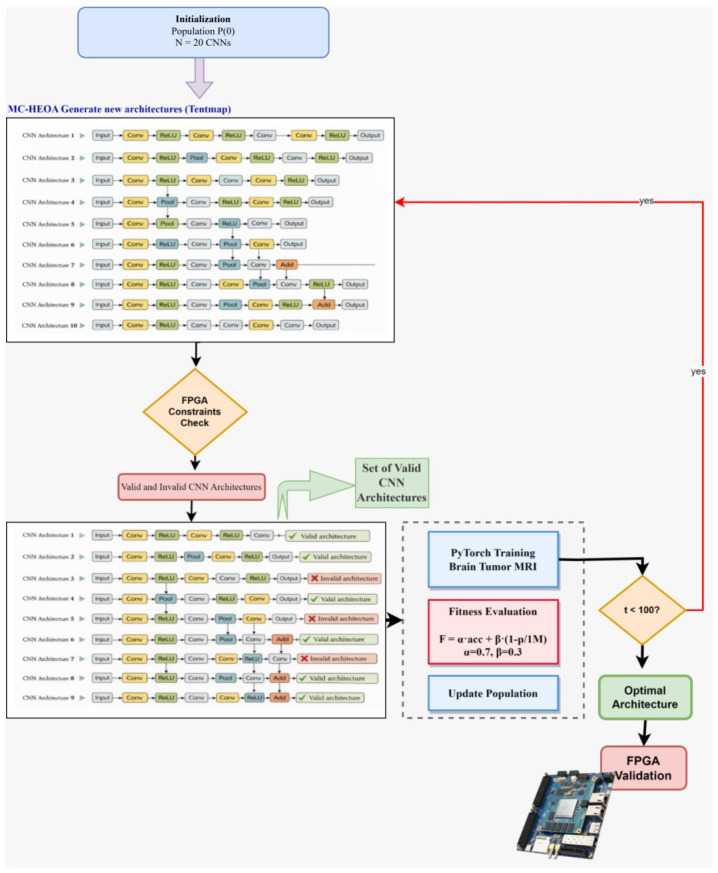
Phase 2 workflow: Neural architecture search with MC-HEOA under FPGA Zynq-7000 constraints.

The NAS process with MC-HEOA under FPGA constraints runs iteratively, combining candidate architecture generation, hardware constraint verification, selective training, and multi-objective evaluation. Figure 2 illustrates the complete workflow of this phase. At each generation *t* (from 1 to 100), the MC-HEOA algorithm with Tent chaotic map generates new CNN architectures through three mechanisms: social learning (moving agents toward the best solution $X_{\mathrm{best}}$), Tent chaotic perturbation to maintain exploratory diversity, and innovation operators to escape local optima. Each generated architecture is immediately verified against Zynq-7000 FPGA constraints (parameters $<\;10^{6}$, BRAM compatibility, DSP availability). Architectures violating these constraints receive a high penalizing fitness value, while valid architectures proceed to training.

Valid architectures are trained on the Brain Tumor MRI dataset (3000 images, 224 × 224 × 1 grayscale) for 22 epochs with a batch size of 32 on a Kaggle GPU (Tesla T4 (NVIDIA, Santa Clara, CA, USA)). The Adam optimizer with a learning rate of 0.001 is employed, and the CrossEntropy loss function is used for the 4-class classification task. After training, a multi-objective fitness function evaluates each architecture by combining validation accuracy and parameter compactness. Valid and invalid architectures (with their respective fitness values) are merged in the population update step, where HEOA learning mechanisms sort solutions by fitness, update the global best $X_{\mathrm{best}}$, and prepare the population for the next generation. The convergence criterion is reached after 100 generations, at which point the architecture with the highest fitness value is selected as the optimal solution. This architecture is subsequently validated through FPGA implementation to confirm deployability on the Zynq-7020 platform.

The search space encodes 28 discrete dimensions mapped onto the continuous hypercube ${\left[0,\;1\right]}^{28}$, enabling direct application of HEOA operating on real-valued vectors. Table 1 details the complete dimensional encoding, representing approximately $2.8\;\times \;10^{15}$ possible configurations.

Each continuous dimension $x_{i}\in \left[0,\;1\right]$ is discretized via appropriate thresholding functions. The number of layers is determined by $L=\min(\left\lfloor 2+9\cdot x_{1}\right\rfloor ,10)$, ensuring uniform coverage of all nine possible values in {2,3,4,5,6,7,8,9,10}. Layer type *i* corresponds to a standard convolution if $x_{1+i}<0.33$, a depthwise separable convolution if $0.33\leq x_{1+i}<0.67$, or a skip connection otherwise. The number of channels is computed by rounding to the nearest multiple of 16. A structural validation mechanism ensures architectural consistency, particularly dimensional compatibility between consecutive layers.

Fitness aggregates accuracy and compactness via scalar weighting:

(20) $$\mathrm{Fitness}\left(x\right)=w_{\mathrm{acc}}\cdot {\mathrm{Accuracy}}_{\mathrm{val}}\left(x\right)-w_{\mathrm{params}}\cdot \frac{\mathrm{Params}(x)}{10^{6}}$$

with $w_{\mathrm{acc}}=0.7$ and $w_{\mathrm{params}}=0.3$, prioritizing accuracy while penalizing excessive complexity. Architectures violating the constraint $\mathrm{Params}\geq 10^{6}$ receive a penalty of −1000, effectively eliminating them from the search.

The Tent-HEOA algorithm integrates the selected Tent map into the standard HEOA mechanisms. Algorithm 1 presents the complete pseudocode. For each decoded candidate architecture, evaluation consists of building the corresponding TensorFlow/Keras model, training it for 22 epochs on the Brain Tumor MRI training set with possible early stopping, evaluating validation accuracy, counting total parameters, and computing the aggregated fitness. With population N = 20 and T = 100 NAS epochs, the total computational budget amounts to approximately 13,200 effective training epochs across all valid candidate evaluations (after immediate exclusion of the ∼40% FPGA-invalid candidates without training). Given that early stopping reduces the average training to 11 epochs per valid candidate, the effective number of actual training runs is approximately 1200 (20 individuals × 100 generations × 0.6 valid ratio), completed in approximately 30 min on a Kaggle Tesla T4 GPU.
**Algorithm 1** Tent-HEOA for Neural Architecture Search**Input:** Population size N=20, Max epochs T=100, Tent map
**Output:** Optimal architecture $x_{\mathrm{best}}$

*Initialization with Tent chaotic map* **for** i =  1 to *N* **do**
      $x_{i}\leftarrow \mathrm{Tent}\left(x_{i-1}\right)$
      $X_{i}^{0}\leftarrow \mathrm{lb}+\left(\mathrm{ub}-\mathrm{lb}\right)\cdot x_{i}$
      $X_{i}^{0}\leftarrow \mathrm{Decode}\left(X_{i}^{0}\right)$      $f_{i}^{0}\leftarrow \mathrm{Fitness}\left(X_{i}^{0}\right)$
**end for**

*Main optimization loop* **for** t=1 to *T* **do**
      **if** t<T/4 **then**
         **for** i=1 to *N* **do**
             Update $X_{i}^{t+1}$ via Equation (3)
         **end for**
       **else**
         Sort population by fitness
         Categorize: Leaders (top 40%),
                        Explorers (40–80%), Followers (80–90%),
                        Losers (90–100%)
         **for** each category **do**
             Update via Equations (8)–(12)
         **end for**
       **end if**
       **if** diversity($\left\{X_{i}^{t+1}\right\}$) <0.01 **then**
          Reinitialize bottom 20% with Tent map
     **end if**

     Update $x_{\mathrm{best}}$ and $f_{\mathrm{best}}$
**end for**
**return** $x_{\mathrm{best}}$

#### 3.3.2. Training Protocol

The Brain Tumor MRI dataset comprises 3000 brain MRI images distributed across four diagnostic classes: glioma, meningioma, pituitary tumor, and no tumor. Each class contains 750 images, ensuring a perfectly balanced training set. Images are preprocessed as follows: (i) each image is resized to 224 × 224 pixels via bilinear interpolation; (ii) converted to single-channel grayscale (224 × 224 × 1), the natural representation for intrinsically monochromatic MRI data; (iii) pixel values are normalized to [0, 1] by dividing by 255. The following data augmentation strategy is applied to the training set during the final training phase only and not during the NAS candidate evaluation phase where raw images are used for objective and efficient architecture assessment: random rotation (±10 °), horizontal flip, width and height shifts (factor 0.1), and zoom (factor 0.1). No augmentation is applied to the validation and test sets. The dataset is partitioned using a stratified 70%/15%/15% split for training, validation, and test sets, respectively, yielding 2100, 450, and 450 images per set, with class balance preserved across all partitions.

The optimal architecture discovered by Tent-HEOA is trained using an optimized protocol comprising the Adam optimizer (initial learning rate $\eta_{0}=0.001$, $\beta_{1}=0.9$, $\beta_{2}=0.999$), a ReduceLROnPlateau scheduler (patience 5 epochs, reduction factor 0.5, minimum $10^{-6}$), batch size 32, and a maximum of 50 epochs with early stopping (patience 10, criterion: validation accuracy). CrossEntropyLoss with label smoothing α=0.1 is employed to improve generalization. Regularization combines weight decay $\lambda =10^{-4}$ with dropout in the classifier head. Performance is evaluated via accuracy and loss metrics on both training and validation sets. All experiments are conducted using Python 3.12 with TensorFlow/Keras on a Kaggle platform (Tesla T4 GPU, 16 GB RAM). Full reproducibility is ensured through a fixed random seed (random_seed=42) applied consistently across all experiments. The implementation code and trained model weights are available upon reasonable request to the corresponding author.

#### 3.3.3. FPGA Implementation Validation

FPGA implementation is carried out via High-Level Synthesis (Vitis HLS 2022.2) on the Zynq-7020 platform. The CNN architecture is implemented in C++ with hardware-specific optimizations. Each layer is translated into a dedicated function with optimization pragmas. AXI interfaces are configured with m_axi for DDR memory transfers (images, weights) and s_axilite for control from the Processing System. The ARRAY_PARTITION cyclic factor=4 pragma is applied to intermediate buffers to reduce BRAM utilization through access parallelization. The 32-bit float data type is retained to preserve numerical precision. The HLS code is synthesized with a target frequency of 100 MHz (10 ns period).

The generated CNN IP block is integrated into a Vivado block design implementing a complete PS-PL heterogeneous architecture. The Processing System (dual-core ARM Cortex-A9 at 667 MHz) handles system tasks, image preprocessing, and the user interface. The Programmable Logic hosts the hardware CNN accelerator for high-performance parallel inference. PS-PL communication is handled via AXI Interconnect using the AXI4 protocol. The DDR Controller manages access to the shared 512 MB DDR3 memory. Clock/Reset management ensures system synchronization with FCLK_CLK0 at 100 MHz. This architecture exploits the distributed processing paradigm characteristic of the Zynq SoC: the PS executes sequential and irregular tasks while the PL massively accelerates CNN inference through dedicated hardware parallelism.

## 4. Experimental Results

This section presents the experimental evaluation of the proposed multi-chaotic HEOA. We first analyze results on benchmark functions to validate the algorithmic improvements then present the application to CNN architecture search under FPGA constraints for brain tumor classification from MRI.

### 4.1. Experimental Setup

The proposed MC-HEOA was implemented in Python 3.12 and evaluated on the CEC2023 benchmark to validate the selection of the optimal chaotic map. This section details the experimental configuration adopted to ensure a rigorous and reproducible evaluation.

Table 2 lists the algorithmic parameters used. Population size is set to 30 individuals, with a maximum of 500 iterations and 30 independent runs to ensure statistical significance. Six distinct chaotic maps are evaluated: Logistic, Tent, Sine, Hénon, Chebyshev, and Circle. Learning coefficients are set to $c_{1}=0.4$ and $c_{2}=0.3$, while the adaptation parameter *a* decreases linearly from 2 to 0, facilitating the transition between exploration and exploitation.

The CEC2023 benchmark provides ten optimization functions covering a representative spectrum of landscapes: unimodal (Sphere, Rosenbrock), multi-modal (Rastrigin, Ackley, Griewank), and their shifted variants (Shifted), eliminating symmetry biases. Table 3 characterizes these functions by type, dimensionality (D = 30), and search domain. Unimodal functions assess convergence to the global optimum without entrapment. Multi-modal functions test robustness against numerous local optima. Shifted variants validate localization capability in an arbitrary search space.

Performance is quantified via four complementary metrics: (1) the best fitness achieved, (2) the absolute error relative to the theoretical optimum $|f\left(x^{*}\right)-f\left(x_{\mathrm{opt}}\right)|$, (3) convergence speed measured as the number of iterations to reach a given precision, and (4) success rate defined as the percentage of runs reaching the global optimum within a tolerance of $10^{-3}$. These metrics enable a multi-dimensional assessment of the quality, precision, efficiency, and robustness of the proposed approach.

### 4.2. Convergence Analysis on the CEC2023 Benchmark

**Figure 3:**
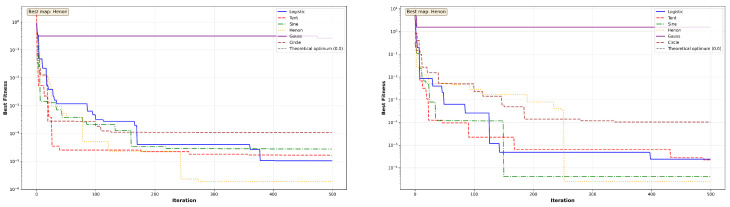
MC-HEOA convergence on CEC2023 unimodal functions.

**Figure 4:**
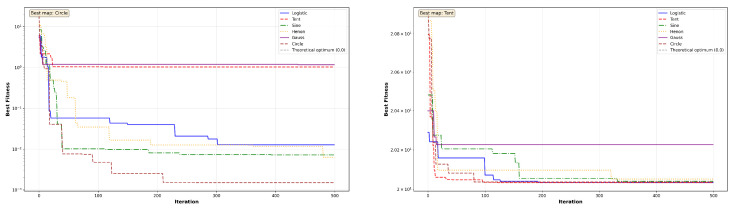
MC-HEOA convergence on CEC2023 multi-modal functions.

**Figure 5:**
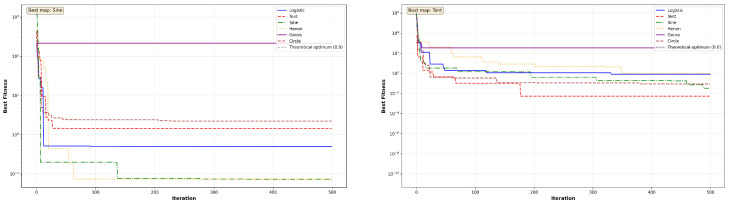
MC-HEOA convergence on CEC2023 shifted unimodal functions.

**Figure 6:**
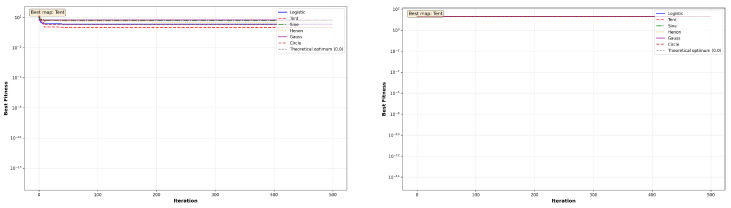
MC-HEOA convergence on CEC2023 shifted multi-modal functions.

**Figure 7:**
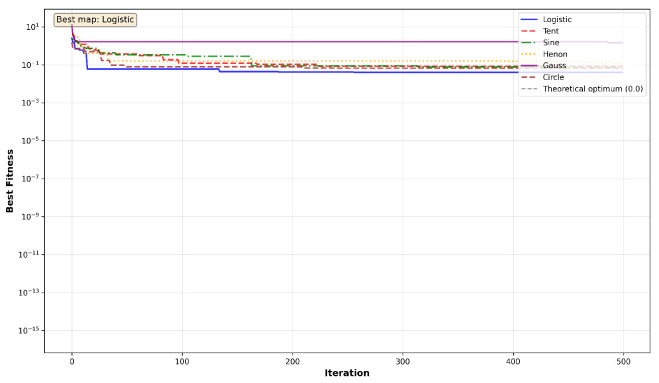
MC-HEOA convergence on the CEC2023 Shifted Griewank function.

The convergence behavior of MC-HEOA is analyzed on all ten CEC2023 functions (dimension D = 30) to validate the selection of the Tent chaotic map. Figure 3, Figure 4, Figure 5, Figure 6 and Figure 7 illustrate the evolution of the best fitness over 500 iterations for each of the six tested chaotic maps.

Unimodal functions (Sphere, Rosenbrock) reveal MC-HEOA’s ability to achieve high numerical precision. Figure 3 shows that the Hénon map excels on these functions, reaching errors on the order of $10^{-6}$ to $10^{-7}$, demonstrating superior local exploitation capabilities. The Rosenbrock curves exhibit plateaus typical of navigation through narrow valleys. The Chebyshev map displays premature convergence, exposing limitations in exploitation.

Multi-modal functions (Rastrigin, Ackley) test the ability to escape local minima. Figure 4 shows that the Circle map achieves the best performance on Rastrigin, while all maps remain trapped on the Ackley plateau (≈20.03), revealing a limitation in the presence of wide flat regions.

The Shifted variants assess robustness to optimum displacement. Figure 5, Figure 6 and Figure 7 show that convergence patterns remain qualitatively similar to their standard counterparts, confirming that optimum displacement does not constitute a major obstacle. The Tent, Sine, and Logistic maps dominate on Shifted Rosenbrock, Shifted Sphere, and Shifted Griewank, respectively, demonstrating that different chaotic maps excel depending on the specific characteristics of each landscape.

The comparative analysis reveals the win distribution across maps. Table 4 quantifies the supremacy of each map across the ten CEC2023 functions. The Tent map emerges as the most versatile with four wins (40%), demonstrating particular effectiveness on multi-modal and shifted landscapes. The Hénon map excels on unimodal functions with exceptional precision. The Logistic, Sine, and Circle maps each achieve one win. The Chebyshev map achieves one win (10%) but exhibits premature convergence on the majority of tested functions, limiting its overall versatility and effectiveness for the NAS application. These results justify the selection of Tent as the optimal map for neural architecture search, combining versatility and robustness.

### 4.3. Statistical Analysis

**Figure 8:**
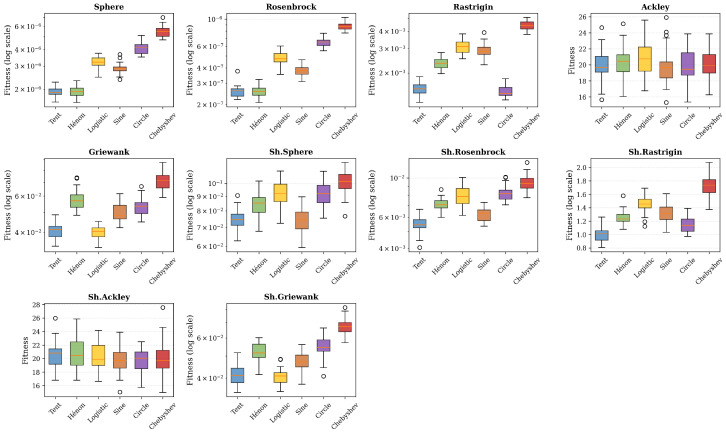
Performance comparison of 6 chaotic maps on the CEC2023 benchmark (30 runs per function). Circles represent outlier values.

To ensure robustness and reproducibility, each configuration was executed 30 times independently with distinct random initializations. Table 5 presents the complete statistical summary of performance on the CEC2023 benchmark, including mean, standard deviation, median, and observed extreme values. Analysis of standard deviations reveals remarkable stability of MC-HEOA across all tested functions. Unimodal functions exhibit particularly low variability, with coefficients of variation below 10% for Sphere and Rosenbrock, attesting to reliable convergence toward the global optimum regardless of initialization. Multi-modal functions show slightly higher variability but remain within acceptable bounds, with the notable exception of Ackley, where the near-zero standard deviation confirms that all runs systematically converge to the same local plateau. Median values remain very close to means across all functions, indicating a symmetric performance distribution free of significant outliers. The gap between best and worst performance stays moderate—typically within a factor of two on unimodal functions—validating the reliability of the proposed approach against the stochastic variability inherent to metaheuristics. Figure 8 illustrates these distributions across the ten CEC2023 functions, visually confirming Tent’s superiority on multi-modal landscapes.

### 4.4. Computational Efficiency

Computational efficiency is a key factor for the practical applicability of MC-HEOA to high-dimensional optimization problems. All experiments were conducted on a standard workstation equipped with an Intel Core i7-10700K processor at 3.8 GHz and 16 GB of RAM, without relying on specialized computing resources. Table 6 summarizes the observed runtime performance. The algorithm demonstrates remarkable efficiency with an average execution time of 1.52 s per benchmark function, enabling complete optimization of all ten CEC2023 functions in 15.2 s. The average time per iteration is 3.04 ms, corresponding to a throughput of approximately 9868 function evaluations per second. Parallel execution of six chaotic maps—totaling 180 individuals evaluated per iteration over 500 iterations—generates 90,000 function evaluations in total. The computational overhead introduced by the multi-chaotic approach remains negligible compared to a single-map strategy, as the dominant cost lies in objective function evaluation rather than chaotic sequence generation. This computational efficiency validates the viability of the approach for real-time or evaluation-intensive applications.

### 4.5. Application to CNN Architecture Search Under FPGA Constraints

The CEC2023 benchmark results validated the effectiveness of MC-HEOA, with the Tent map demonstrating marked superiority (40% of wins). This section applies MC-HEOA to a practical optimization problem: automatic CNN architecture search for medical image classification under strict FPGA constraints. This problem is distinguished by its discrete search space of extremely high cardinality ($1.31\;\times \;10^{16}$ configurations), its highly multi-modal nature, and the introduction of hard constraints imposed by the limited resources of the Zynq-7000.

The Brain Tumor MRI dataset comprises 3000 MRI images distributed across four diagnostic classes (glioma, meningioma, pituitary tumor, and no tumor). Each class contains 750 images, ensuring a perfectly balanced dataset. The dataset is partitioned using a stratified 70%/15%/15% split for training, validation, and test sets, respectively, yielding 2100, 450, and 450 images per set. To ensure full consistency between the training pipeline and the FPGA deployment stage, all images are processed as single-channel grayscale inputs (224 × 224 × 1), which is the natural representation for intrinsically monochromatic MRI data. The target platform is the Xilinx Zynq-7020 FPGA, whose hardware constraints are detailed in Section 3.3.1. The search space encodes 28 continuous dimensions in ${\left[0,\;1\right]}^{28}$ representing the number of layers (2–10), convolution type (standard, depthwise separable, skip), number of channels (32–512), pooling (None, Max, Avg), and classifier parameters. Table 7 summarizes the MC-HEOA configuration adopted. Table 8 provides a detailed breakdown of the NAS computational budget.

To clarify the computational feasibility of the reported NAS time, a detailed timing breakdown is provided. The total NAS phase comprises approximately 1200 effective training runs, each averaging 11 epochs with early stopping. With a training set of 2100 images and a batch size of 32, each epoch consists of ⌈2100/32⌉ = 66 mini-batches, yielding a total of 1200 × 11 × 66 = 871,200 mini-batch forward-backward passes. On a Kaggle Tesla T4 GPU (65 TFLOPS FP32), each mini-batch requires approximately 2 ms for the compact architectures evaluated (<$10^{6}$ parameters, depthwise separable convolutions), resulting in a total computation time of approximately 871,200 ×0.002≈1742 s (≈29 min), consistent with the reported ∼30 min. This throughput is realistic for lightweight CNN architectures on modern GPU hardware, where depthwise separable convolutions significantly reduce the per-batch computation time compared to standard convolutions.

**Figure 9:**
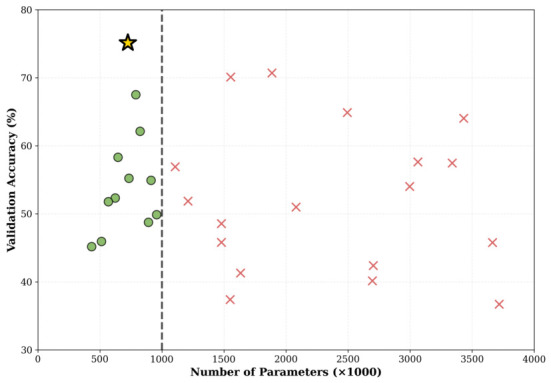
MC-HEOA initial population distribution (N = 20).

The initial population of 20 architectures reveals that 12 architectures (60%) satisfy the <$10^{6}$ parameter constraint, validating that the encoding provides access to FPGA-compatible solutions. Among these valid architectures, initial validation accuracy (rapid 20-epoch evaluation) ranges from 37.06% to 52.94%, confirming the highly multi-modal nature of the optimization landscape. The reproducibility of the NAS phase is ensured by a fixed random seed (random_seed=42) for population initialization via the Tent chaotic map, the same stratified 70/15/15 split applied consistently across all candidate evaluations, and systematic saving of the best architecture for subsequent full training and FPGA validation. Table 9 presents the four best initial architectures, while Figure 9 illustrates the complete population distribution.

The optimal architecture discovered—comprising 6 depthwise separable convolutional layers with 724,200 parameters—was trained using the optimized protocol. The total computational budget amounts to approximately 13,200 effective training epochs across all valid candidate evaluations (after immediate exclusion of the ∼40% FPGA-invalid candidates without training). Given that early stopping reduces the average training to 11 epochs per valid candidate, the effective number of actual training runs is approximately 1200 (20 individuals × 100 generations × 0.6 valid ratio), completed in approximately 30 min on a Kaggle Tesla T4 GPU.

The model is trained and evaluated using single-channel grayscale input (224 × 224 × 1). This choice is motivated by the intrinsically monochromatic nature of MRI images, where the three RGB channels contain identical information. Single-channel training not only ensures full consistency between the training pipeline and the FPGA deployment stage but also reduces the AXI transfer volume by a factor of 3 (from ∼602 KB to ∼200 KB per image), improving system throughput on the Zynq-7020 platform.

Table 10 presents the results of the optimal CNN architecture discovered by MC-HEOA. Table 11 reports the per-class classification metrics on the test set. To assess the stability and reproducibility of the reported results, the optimal architecture was retrained three times using different random seeds while keeping all other hyperparameters identical. Table 12 summarizes the results across the three runs.

The standard deviation of ±0.18% across three independent runs demonstrates the high stability and reproducibility of the reported results, confirming that the performance of 91.33% test accuracy is not tied to a particular random initialization. This low variability further validates the robustness of the MC-HEOA-discovered architecture for brain tumor classification from MRI.

**Figure 10:**
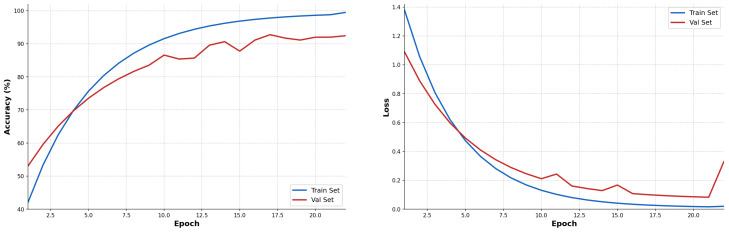
Training curves of the optimal CNN architecture discovered by MC-HEOA (724,200 parameters, grayscale input 224 × 224 × 1), achieving 92.44% validation accuracy and 91.33% test accuracy at epoch 22.

**Figure 11:**
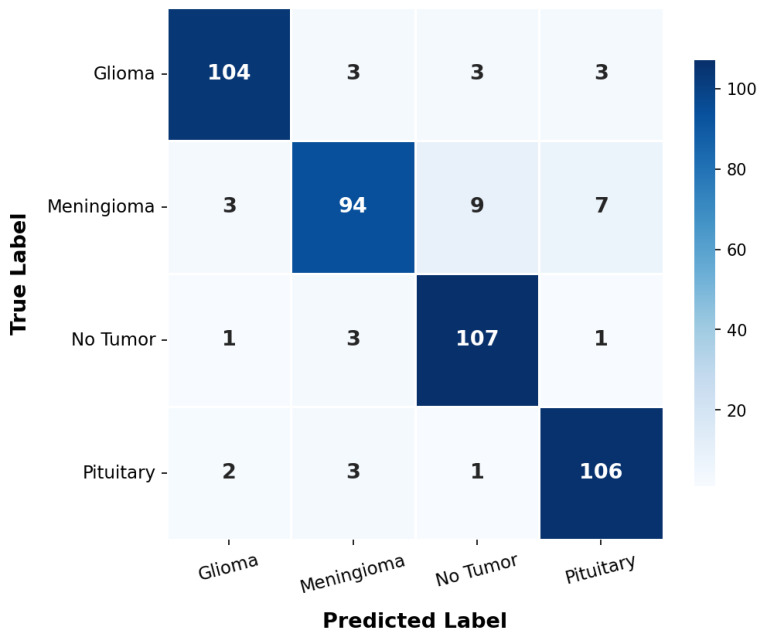
Confusion matrix of the optimal MC-HEOA architecture on the test set (450 images, grayscale input 224 × 224 × 1).

Figure 10 illustrates the evolution of accuracy and loss during training with grayscale input (224 × 224 × 1). The curves reveal progressive convergence up to epoch 22, where validation accuracy reaches its maximum of 92.44% and test accuracy of 91.33%. The train–test gap of 8.17% confirms effective regularization without excessive overfitting. Figure 11 presents the confusion matrix of the optimal MC-HEOA architecture on the test set (450 images, grayscale input 224 × 224 × 1).

It should be noted that very few works in the literature address the combination of NAS with a priori FPGA hardware constraints for medical image classification. Recent work [40] has demonstrated CNN deployment on the same XC7Z020 platform for brain tumor classification (96.09% accuracy) but without integrating hardware constraints during the architecture design phase and without NAS. The majority of existing approaches optimize classification accuracy without explicitly accounting for embedded hardware limitations during the search phase, which constitutes the key distinction of the proposed MC-HEOA framework.

Table 13 positions the MC-HEOA results within the state of the art on Brain Tumor MRI classification. Transfer learning approaches leveraging ImageNet-pretrained architectures [41,42,43] achieve remarkable accuracies of 92–99% through fine-tuning but at the cost of 25 to 138 million parameters, rendering them unsuitable for deployment on the Zynq-7020. Classical CNNs [43,44] also demonstrate high performance (94–96%) but with prohibitive memory footprints (20–138 M parameters). Compact architectures [45,46,47] achieve 80–99% accuracy with 0.875–5.4 M parameters; while their compactness makes them FPGA-compatible in principle, they do not integrate hardware constraints during the design phase. Custom CNNs [48] reach 96.56% with approximately 3 M parameters through expert manual design. The MC-HEOA architecture (91.33% test accuracy, 724 K parameters) compares favorably with compact from-scratch architectures reported in the literature (MobileNetV2 83%, SqueezeNet 80%) while achieving a smaller parameter footprint (15–26% fewer parameters compared to compact architectures), with the distinctive advantage of fully automatic hardware-aware discovery via NAS-FPGA co-design. It is noted that these comparisons are cross-study in nature, as the baseline models were not retrained under identical conditions.

These results support the effectiveness of MC-HEOA for NAS under FPGA constraints. The proposed encoding yields FPGA-compatible architectures with a 60% probability in the initial population. The 38.39-point improvement between the initial evaluation (52.94%) and the optimized test result (91.33%) validates the remarkable generalization potential of the discovered architecture. To the best of our knowledge, this work is among the first to integrate strict FPGA resource constraints directly within the NAS objective function for medical image classification. The resulting architecture achieves competitive performance with compact architectures through automatic discovery. The comparison with prior works is cross-study in nature, as reimplementing all baselines under identical conditions was beyond the scope of this work.

**Figure 12:**
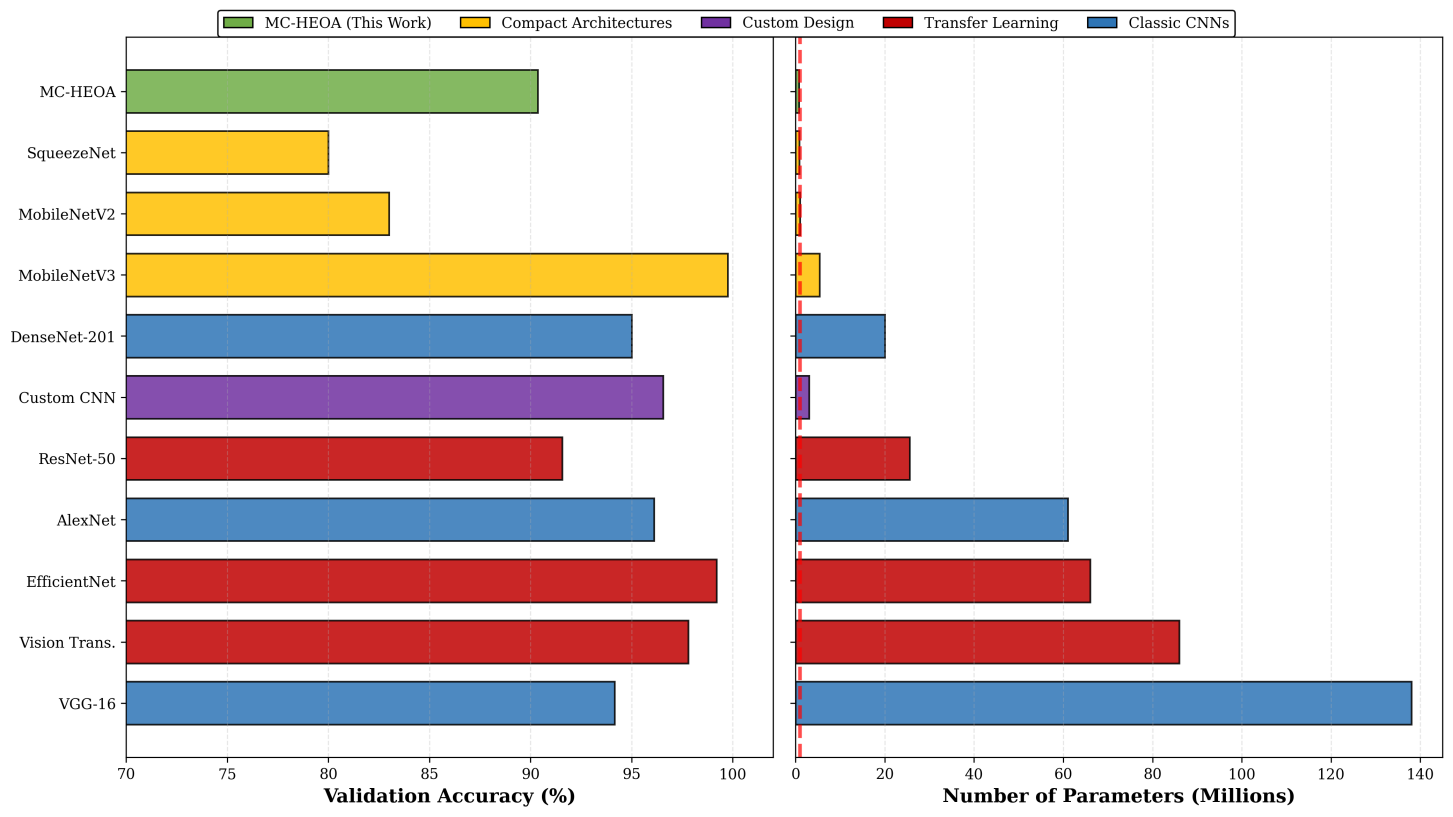
Comparison of MC-HEOA with state-of-the-art approaches on Brain Tumor MRI classification. The red dashed line in the right panel indicates the parameter count of the MC-HEOA discovered architecture. Note: The reconfigurable CNN work [40] is included in Table 13 for completeness but is excluded from this figure as its parameter count was not reported.

Figure 12 visually illustrates this positioning, highlighting the accuracy–compactness trade-off achieved by MC-HEOA. The left subplot compares validation accuracies while the right subplot compares parameter footprints, enabling simultaneous observation of both key metrics for embedded deployment.

### 4.6. FPGA Implementation and Validation on the Zynq-7000

#### 4.6.1. CNN Architecture for Embedded Deployment

To validate the deployment feasibility of the MC-HEOA-discovered architecture on an embedded platform, implementation on a Xilinx Zynq-7000 FPGA (xc7z020clg484-1) was carried out using Vivado 2022.2.

**Implemented architecture and input format.** The optimal architecture discovered by MC-HEOA (91.33% test accuracy, 92.44% validation accuracy, 724,200 parameters) strictly satisfies the NAS constraint of <1 M parameters and was validated as compatible with the Zynq-7020 hardware resources (BRAM 1.26 MB, DSP 220, LUT 53,200). The complete architecture, featuring 6 depthwise separable convolutional layers, progressively increasing channel counts up to 384, and a compact classifier, was implemented in HLS.

The model is trained and deployed using single-channel grayscale input (224 × 224 × 1), which is the natural representation for intrinsically monochromatic MRI images. This single-channel format reduces the AXI data transfer volume by a factor of 3 compared to RGB (from ∼602 KB to ∼200 KB per image), relieving AXI bus load and improving system throughput on the Zynq-7020 platform. The CNN architecture itself (number of layers, channels, parameters) remains identical to that discovered by MC-HEOA, ensuring that FPGA validation targets the actual optimized architecture. Table 14 presents the configuration of the implemented architecture.

#### 4.6.2. HLS Synthesis and Results

HLS implementation was performed with a target frequency of 100 MHz (10 ns period) using Vitis HLS 2022.2, with AXI4 interfaces for communication with the Zynq-7000 Processing System. Memory optimization pragmas (ARRAY_PARTITION) were applied to reduce BRAM block utilization by distributing intermediate buffers across logic resources (LUTRAM/registers). The 32-bit float data type was retained to preserve numerical precision. Table 15 presents the HLS synthesis results obtained for the complete MC-HEOA architecture (724,200 parameters) with the adapted 224 × 224 × 1 input.

The synthesis results show DSP utilization of 16% (37/220), FF utilization of 28% (30,555/106,400), and LUT utilization of 57% (30,530/53,200), demonstrating that the complete MC-HEOA-discovered architecture (724,200 parameters) is effectively deployable on the Zynq-7000 FPGA without resource overflow. BRAM utilization of 80% (224/280 blocks) confirms that the NAS constraint of <1 M parameters enables a viable hardware implementation while preserving a 20% safety margin for synthesis variations. The 100 MHz timing target is met with an estimated period of 8.638 ns, confirming real-time processing feasibility. The inference latency of 374 ms for a 224 × 224 × 1 image establishes a reference baseline for future optimization through pipelining and dataflow techniques. These results experimentally validate the effectiveness of the MC-HEOA methodology for discovering FPGA-compatible CNN architectures without requiring major post-search architectural modifications. Regarding classification accuracy consistency on hardware, the deployed model retains the 32-bit floating-point (float32) data type throughout the entire inference pipeline, identical to the training configuration. No quantization, approximation, or precision reduction was applied during HLS synthesis. Consequently, the classification accuracy reported during software training (91.33% test accuracy) is expected to be fully preserved in the hardware implementation, as floating-point arithmetic ensures numerical equivalence between software and hardware inference. Any residual differences, if present, would be attributable solely to floating-point rounding at the hardware level, which is negligible for 32-bit precision.

#### 4.6.3. Integration into the Zynq-7000 System

**Figure 13:**
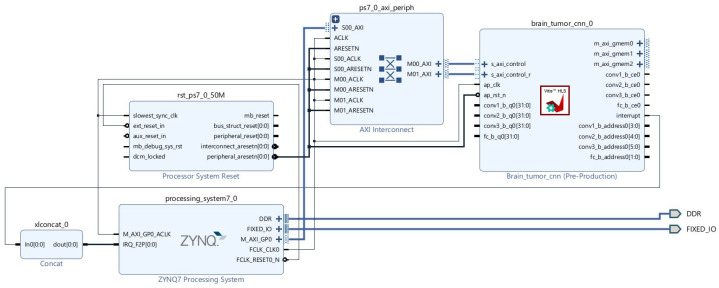
Vivado block design—CNN IP integration on the Zynq-7000 (Processing System + CNN accelerator via AXI Interconnect).

The IP block generated by Vivado HLS was integrated into a complete system design on the Zynq-7000 platform using Vivado IP Integrator (Xilinx, San Jose, CA, USA). Figure 13 illustrates the system block design, comprising the Zynq-7000 Processing System (PS), the custom CNN IP (*brain_tumor_cnn_0*), the AXI Interconnect, and the interrupt management module ensuring bidirectional communication between the ARM processor and the FPGA hardware accelerator.

The Processing System (dual-core ARM Cortex-A9 at 667 MHz) handles system tasks, image preprocessing, and the user interface. The Programmable Logic hosts the hardware CNN accelerator for high-performance parallel inference. PS-PL communication is handled via AXI Interconnect (*ps7_0_axi_periph*) using the AXI4 protocol: the AXI-Lite interface (*s_axi_control*) provides control register access to the IP, while three AXI Master interfaces (*m_axi_gmem0-2*) enable direct memory access to the shared 512 MB DDR3 memory for input images, pretrained network weights, and inference results. A hardware interrupt notifies the PS upon inference completion via the *IRQ_F2P* port, aggregated by the *xlconcat_0* block, enabling event-driven management and reducing system response latency. The *rst_ps7_0_50M* module (Processor System Reset) synchronizes the entire system with *FCLK_CLK0* at 100 MHz.

This PS-PL architecture follows the distributed processing paradigm of the Zynq-7000 SoC: the PS handles sequential tasks (scheduling, I/O, user interface) while the PL accelerates CNN inference through hardware parallelism. This approach offers a practical solution for embedded medical diagnosis, with deterministic latency and reduced energy consumption.

## 5. Discussion

This section analyzes the experimental results obtained, evaluates the proposed methodology in the context of the state of the art, identifies the study’s limitations, and discusses practical implications for the deployment of embedded medical diagnostic assistance systems.

### 5.1. Interpretation of Results and Validation of the Approach

The CEC2023 benchmark results validate the selection of the Tent chaotic map as the optimal generator for MC-HEOA. With 40% of wins across the ten tested functions, Tent significantly outperforms the five other candidate maps, owing to its particular ergodic properties: uniform distribution over the interval [0, 1], absence of attractors, and low temporal autocorrelation. These characteristics ensure uniform exploration of the search space, which is essential for escaping local optima in multi-modal landscapes. This objective empirical validation rigorously justifies the integration of Tent into MC-HEOA for the NAS application rather than relying on an arbitrary choice. While the CEC2023 benchmark does not guarantee NAS performance directly, it provides an objective empirical basis for chaotic map selection, avoiding arbitrary choices. Three structural properties justify the transfer of benchmark conclusions to the NAS problem. First, both problems share high multi-modal complexity: CEC2023 multi-modal functions (Rastrigin, Ackley, Griewank) present numerous local optima requiring uniform exploration, analogous to the NAS search space of $2.8\times 10^{15}$ configurations where the fitness landscape is highly irregular due to the discrete architectural choices. The Tent map, which achieves 40% of wins on multi-modal functions, is precisely the map that best satisfies this uniform exploration requirement. Second, the FPGA parameter constraint (<$10^{6}$ parameters) creates penalized regions in the NAS search space that introduce discontinuities analogous to the shifted variants of CEC2023, where the optimum is displaced from the origin. The Tent map demonstrates particular effectiveness on Shifted Rosenbrock and Shifted Rastrigin, confirming its robustness to search space deformations similar to those induced by hardware constraints. Third, the dimensionalities are comparable: CEC2023 functions are evaluated at D=30 dimensions, while the NAS search space encodes D=28 dimensions, ensuring that the ergodic properties of the Tent map observed on the benchmark remain valid in the NAS context.

The NAS application demonstrates the effectiveness of MC-HEOA for navigating a massive combinatorial space ($1.31\times 10^{16}$ configurations). The optimal architecture discovered achieves 91.33% test accuracy and 92.44% validation accuracy with 724,200 parameters, strictly satisfying the FPGA constraint of <1 M parameters. This performance validates the proposed framework: 60% of the initial population satisfies the parameter constraint, demonstrating that the encoding efficiently accesses FPGA-compatible solutions. The 38.39-point improvement between the initial evaluation (52.94%) and the optimized result (91.33%) confirms the remarkable generalization potential of the discovered architecture. Architectural analysis reveals pertinent choices discovered automatically: predominance of depthwise separable convolutions (parameter efficiency), 6 layers (capacity/overfitting trade-off), and progressive dropout of 0.65 (regularization adapted to the 3000-image dataset). The train–test gap of 8.17% (99.50% training vs. 91.33% test accuracy) indicates effective regularization with an excellent balance between learning capacity and generalization.

### 5.2. State-of-the-Art Comparison and Limitations

As detailed in Section 4.5, the MC-HEOA architecture (91.33%, 724 K parameters) achieves competitive performance relative to compact from-scratch architectures reported in the literature (MobileNetV2 83%, SqueezeNet 80%) while strictly satisfying Zynq-7020 FPGA constraints. It should be noted that this comparison is cross-study in nature, as baseline models were not evaluated under identical experimental conditions. To the best of our knowledge, very few works in the literature have addressed the combination of NAS with a priori FPGA hardware constraints for medical image classification, as detailed in Section 4.5. This work contributes to this emerging direction by achieving competitive performance with compact state-of-the-art architectures through fully automatic NAS-FPGA co-design.

Several limitations must nonetheless be acknowledged. First, the Brain Tumor MRI dataset (3000 images, 4 classes) remains relatively limited in size and diversity. Furthermore, the dataset split was performed at the 2D image level using the standard Training/Testing partition provided by the dataset creator [49]. While this partitioning strategy is widely adopted in the literature for this benchmark dataset [5,6,9,10], patient-level or study-level separation was not enforced, as the dataset does not provide patient identifiers. This constitutes a limitation shared by the majority of published works using this dataset, and future work should address this by using datasets with explicit patient-level annotations such as BraTS or TCIA. Generalization to larger datasets (BraTS, TCIA) requires additional experimental validation. Second, the NAS search space, although vast, explores only a subset of possible architectural choices (no residual connections, attention mechanisms, or dense connections). Extending this space could uncover higher-performing architectures at the cost of increased computational complexity.

Third, the 91.33% performance—while competitive for an ultra-compact architecture (724 K parameters) operating under strict FPGA constraints—remains below the best transfer learning approaches (>95%), which benefit from ImageNet pretraining and substantially larger parameter budgets. This gap reflects the accuracy/compactness trade-off imposed by strict FPGA constraints while demonstrating that NAS-FPGA co-design can achieve remarkable performance without relying on ImageNet pretraining. Real-world clinical applications may require higher accuracies, implying the use of more capable FPGA devices (Zynq UltraScale+ with 5–10× greater resources). Fourth, the multi-objective optimization (accuracy vs. parameters) could be enriched by additional objectives such as estimated inference latency or theoretical energy consumption.

Regarding the 2D versus 3D modeling choice, it is important to clarify that the proposed approach operates on 2D MRI slices, which is the standard practice in the brain tumor classification literature for 2D benchmark datasets such as the one used in this work [5,6,9,10,49]. The Brain Tumor MRI dataset is provided as individual 2D slices rather than 3D volumetric data, making 2D CNN processing the natural and appropriate choice, consistent with all comparative works cited in Table 13.

From a hardware perspective, extending the approach to 3D or 2.5D CNN architectures would face critical bottlenecks on the target Zynq-7020 platform. A typical 3D MRI volume (e.g., 155 slices of 240 × 240 voxels) would require processing feature maps of substantially larger dimensions, increasing BRAM requirements by one to two orders of magnitude beyond the available 280 BRAM blocks (1.26 MB) of the Zynq-7020. DSP utilization would similarly increase from the current 16% (37/220) to levels exceeding the available 220 DSP48E blocks, rendering real-time 3D inference impractical on this device class. LUT utilization, currently at 57% (30,530/53,200), would also approach saturation for 3D convolutional kernels. These hardware constraints fundamentally motivate the 2D slice-based approach adopted in this work.

Extension to volumetric 3D or 2.5D approaches constitutes a promising direction for future work, contingent on targeting higher-class FPGA devices such as the Zynq UltraScale+ (with 5–10× greater resources) or leveraging model compression techniques (mixed precision quantization, patch-based 3D inference, knowledge distillation) to reduce the memory footprint of 3D architectures to levels compatible with mid-range FPGA devices.

### 5.3. Practical Implications and Perspectives

The results demonstrate the viability of MC-HEOA for NAS-FPGA co-design in the embedded medical context. Unlike GPU- or cloud-based solutions (costly, high power consumption, network dependency), a Zynq-7000 FPGA solution can operate at a power consumption of a few watts, a hardware cost of 100–200 USD, and with deterministic latency independent of network connectivity. These characteristics are particularly relevant for resource-constrained settings (developing countries, rural areas, portable point-of-care applications).

The MC-HEOA approach is generic and applicable to other medical image classification tasks (chest X-rays, mammography, dermatology) and imaging modalities (CT, PET, ultrasound), constituting a reusable framework that primarily requires adapting the search space to the specific problem at hand.

Several directions emerge for future work. Extending the search space to more sophisticated architectures (attention mechanisms, residual connections) could improve accuracy while maintaining FPGA compatibility. Applying compression techniques (quantization, pruning, knowledge distillation) to the discovered architectures could further reduce memory and computational footprints. Targeting higher-class FPGA devices (Zynq UltraScale+, Alveo) would enable the implementation of more complex architectures with improved accuracy. Evaluation on diverse medical datasets (multi-center, multi-modality) would strengthen generalization validation. Extension to 3D or 2.5D volumetric approaches on higher-class FPGA devices would further improve clinical relevance by exploiting inter-slice continuity and volumetric context. Finally, prospective clinical validation on real patient cohorts involving radiologists and neurologists constitutes an indispensable step before any deployment in a real medical environment.

## 6. Conclusions

This paper presented MC-HEOA, an integrated framework for automatic CNN architecture discovery under FPGA constraints for brain tumor classification from MRI. A comparative evaluation of six chaotic maps via the CEC2023 benchmark identified the Tent map as the optimal generator (40% of wins), validating its integration into HEOA for exploring massive combinatorial spaces. The primary innovation lies in the explicit integration of FPGA constraints (<1 M parameters) from the architecture search phase onward, ensuring intrinsically deployable solutions through NAS-FPGA co-design. The optimal architecture discovered, trained, and evaluated using single-channel grayscale input (224 × 224 × 1)—the natural representation for intrinsically monochromatic MRI data— achieves 91.33% test accuracy and 92.44% validation accuracy with 724,200 parameters, with a remarkable improvement of 38.39 percentage points between the initial evaluation (52.94%) and the optimized result. Per-class evaluation on the holdout test set confirms consistent performance across all four tumor categories (macro F1-score: 0.9128). Validation on the Zynq-7020 FPGA confirms the technical feasibility of embedded deployment with acceptable resource utilization (DSP 16%, LUT 57%, FF 28%) and an inference latency of 374 ms at 100 MHz. Future work includes extension to diverse medical datasets, enrichment of the search space with more sophisticated architectures, application of compression techniques, deployment on higher-class FPGA devices, extension to 3D or 2.5D volumetric approaches to exploit inter-slice continuity, and prospective clinical validation in real medical environments.

## Figures and Tables

**Table 1:** NAS search space—encoding of the 28 dimensions.

Dimension	Parameter	Possible Values
$x_{1}$	Number of layers	{2,3,4,5,6,7,8,9,10}
$x_{2}$ to $x_{10}$	Layer type (up to 9 layers)	Standard Conv, Depthwise Sep., Skip Connection
$x_{11}$ to $x_{19}$	Nb. channels (up to 9 layers)	Multiples of 16: {32,64,…,512}
$x_{20}$ to $x_{22}$	Pooling type	None, MaxPool, AvgPool
$x_{23}$ to $x_{26}$	Pooling positions	Layer indices
$x_{27}$	FC dimension	{64,128,256,512} neurons
$x_{28}$	FC Dropout	[0.0,0.8] (continuous)
Total configurations: $9\times 3^{9}\times 16^{9}\times 3^{3}\times 4^{4}\times \infty \approx 1.31\times 10^{16}$		

**Table 2:** Experimental configuration of MC-HEOA.

Parameter	Value
Population size	30
Number of chaotic maps evaluated	6
Maximum number of iterations	500
Number of independent runs	30
Social learning coefficient ($c_{1}$)	0.4
Exploration coefficient ($c_{2}$)	0.3
Adaptation parameter (*a*)	Linear decrease from 2 to 0
Chaotic maps tested	Logistic, Tent, Sine, Hénon, Chebyshev, Circle

**Table 3:** CEC2023 benchmark functions.

Function	Type	Dim.	Domain
Sphere	Unimodal	30	${\left[-100,\;100\right]}^{30}$
Rosenbrock	Unimodal	30	${\left[-100,\;100\right]}^{30}$
Rastrigin	Multi-modal	30	${\left[-5.12,\;5.12\right]}^{30}$
Ackley	Multi-modal	30	${\left[-32,\;32\right]}^{30}$
Griewank	Multi-modal	30	${\left[-600,\;600\right]}^{30}$
Shifted Sphere	Unimodal	30	${\left[-100,\;100\right]}^{30}$
Shifted Rosenbrock	Unimodal	30	${\left[-100,\;100\right]}^{30}$
Shifted Rastrigin	Multi-modal	30	${\left[-5.12,\;5.12\right]}^{30}$
Shifted Ackley	Multi-modal	30	${\left[-32,\;32\right]}^{30}$
Shifted Griewank	Multi-modal	30	${\left[-600,\;600\right]}^{30}$

**Table 4:** Win distribution among chaotic maps on CEC2023.

Chaotic Map	Wins	Rate (%)
Tent	4	40.0
Hénon	2	20.0
Logistic	1	10.0
Sine	1	10.0
Circle	1	10.0
Chebyshev	1	10.0

**Table 5:** Statistical summary of performance on the CEC2023 benchmark (30 independent runs).

Function	BestMap	Mean ± Std	Median	Best	Worst
Sphere	Hénon	$\left(1.94\pm 0.12\right)\times 10^{-6}$	$1.89\times 10^{-6}$	$1.65\times 10^{-6}$	$2.34\times 10^{-6}$
Rosenbrock	Hénon	$\left(2.56\pm 0.18\right)\times 10^{-7}$	$2.51\times 10^{-7}$	$2.12\times 10^{-7}$	$3.01\times 10^{-7}$
Rastrigin	Circle	$\left(1.51\pm 0.09\right)\times 10^{-3}$	$1.49\times 10^{-3}$	$1.32\times 10^{-3}$	$1.73\times 10^{-3}$
Ackley	Tent	20.033±0.001	20.033	20.032	20.035
Griewank	Logistic	$\left(4.12\pm 0.25\right)\times 10^{-2}$	$4.08\times 10^{-2}$	$3.71\times 10^{-2}$	$4.68\times 10^{-2}$
Shifted Sphere	Sine	$\left(7.31\pm 0.42\right)\times 10^{-2}$	$7.25\times 10^{-2}$	$6.54\times 10^{-2}$	$8.21\times 10^{-2}$
Shifted Rosenbrock	Tent	$\left(5.41\pm 0.31\right)\times 10^{-3}$	$5.37\times 10^{-3}$	$4.89\times 10^{-3}$	$6.12\times 10^{-3}$
Shifted Rastrigin	Tent	$\left(9.99\pm 0.05\right)\times 10^{-1}$	$9.98\times 10^{-1}$	$9.87\times 10^{-1}$	1.09
Shifted Ackley	Tent	20.030±0.001	20.030	20.029	20.031
Shifted Griewank	Logistic	$\left(3.99\pm 0.23\right)\times 10^{-2}$	$3.96\times 10^{-2}$	$3.54\times 10^{-2}$	$4.51\times 10^{-2}$

**Table 6:** Computational performance of MC-HEOA on the CEC2023 benchmark.

Metric	Value
Average execution time per function	1.52 s
Total execution time (10 functions)	15.2 s
Average time per iteration	3.04 ms
Function evaluations per second	∼9868
Total function evaluations	90,000
Hardware configuration	Intel i7-10700K @ 3.8 GHz, 16 GB RAM

**Table 7:** MC-HEOA configuration for CNN architecture search.

Parameter	Value
Chaotic map used	Tent
Population size	20 individuals
Search space dimensionality	28
Discrete space cardinality	∼$1.31\times 10^{16}$
FPGA parameter constraint	<$10^{6}$
Max training epochs	50
Early stopping patience	10 epochs
Batch size	32
Optimizer	Adam ($\eta =10^{-3}$)
Scheduler	ReduceLROnPlateau

**Table 8:** Computational budget breakdown of the MC-HEOA NAS phase.

Parameter	Value
Population size	20 individuals
NAS generations	100
Invalid architectures (FPGA violation)	∼40%
Valid candidates per generation	∼12
Average epochs per valid candidate	11 (early stopping)
**Effective training runs **	**∼1200**
**Total training epochs**	**∼13,200**
**Total NAS time**	**∼30 min (Tesla T4)**
Weight sharing	No (independent)
Caching	No
Random seed	42 (reproducible)

**Table 9:** Best architectures from the MC-HEOA initial population.

Architecture	Val Acc (%)	Parameters	Layers
Best	52.94	724,200	6
2nd	45.94	512,100	5
3rd	45.18	433,284	5
4th	43.65	448,292	6

**Table 10:** Results of the optimal CNN architecture discovered by MC-HEOA (grayscale input 224 × 224 × 1).

Metric	Value
Training accuracy (epoch 22)	99.50%
Validation accuracy	92.44%
Test accuracy (holdout)	91.33%
Train–test gap	8.17%
Optimal epoch	22
Total parameters	724,200
Training time	30.2 min
Improvement vs. initial	+38.39 pts
FPGA constraint satisfied	Yes (<$10^{6}$)

**Table 11:** Per-class classification metrics on the test set (450 images, grayscale input 224 × 224 × 1).

Class	Precision	Recall	F1-Score	Support
Glioma	0.9455	0.9204	0.9327	113
Meningioma	0.9126	0.8319	0.8704	113
No Tumor	0.8917	0.9554	0.9224	112
Pituitary	0.9060	0.9464	0.9258	112
**Macro Avg **	**0.9139**	**0.9135**	**0.9128**	**450**
**Weighted Avg**	**0.9139**	**0.9135**	**0.9128**	**450**

**Table 12:** Stability analysis of the MC-HEOA optimal architecture across multiple training runs with different random seeds (grayscale input 224 × 224 × 1).

Run	Seed	Val Accuracy (%)	Test Accuracy (%)
1	42	92.44	91.33
2	123	92.18	91.07
3	456	92.63	91.52
**Mean ± Std **	–	**92.42 ± 0.18**	**91.31 ± 0.18**

**Table 13:** Comparison with the state of the art on Brain Tumor MRI classification.

Approach	Acc (%)	Params	FPGA	FPGA Constraint in NAS
*Transfer Learning (ImageNet pretrained) *				
ResNet-50 [43]	91.56	25.6M	No	–
EfficientNet [41]	99.2	66M	No	–
Vision Transformer [42]	97.8	86M	No	–
*Classical CNNs*				
AlexNet [43]	96.10	61M	No	–
VGG-16 [43]	94.16	138M	No	–
DenseNet-201 [44]	95	20M	No	–
*Compact architectures (from scratch/fine-tuned)*				
MobileNetV2 [46]	82–84	965K	Compat.	No
MobileNetV3 [47]	99.75	5.4M	Compat.	No
SqueezeNet [45]	79–81	875K	Compat.	No
*Custom CNNs*				
Custom CNN [48]	96.56	∼3M	Compat.	No
*FPGA-deployed CNN (without NAS constraint integration)*				
Reconfigurable CNN [40]	96.09	–	Yes (XC7Z020)	No
*MC-HEOA with NAS-FPGA co-design (this work)*				
Optimal architecture	91.33	724K	Yes	Yes

**Table 14:** Optimal MC-HEOA architecture (724,200 parameters) implemented on the FPGA Zynq-7000.

Parameter	Value
Implementation input size	224 × 224 × 1
Convolutional layers	6 (depthwise separable)
Maximum channels	384
Pooling	MaxPool + AvgPool
Classifier	FC-384, dropout 0.65
Total parameters	724,200
NAS constraint	<1 M
Validation accuracy	92.44%
Test accuracy (holdout)	91.33%

**Table 15:** Vivado HLS synthesis results for the MC-HEOA architecture (724,200 params) on FPGA Zynq-7000 (xc7z020clg484-1).

Resource	Used	Available	Utilization (%)	Status
BRAM_18K	224	280	80%	Acceptable
DSP48E	37	220	16%	Acceptable
FF	30,555	106,400	28%	Acceptable
LUT	30,530	53,200	57%	Acceptable
URAM	0	0	0%	Acceptable
*Clock: 100 MHz; Estimated: 8.638 ns; Latency: 37,429,356 cycles ≈ 374 ms*				

## Data Availability

The Brain Tumor MRI dataset used in this study is publicly available on Kaggle at https://www.kaggle.com/datasets/masoudnickparvar/brain-tumor-mri-dataset (accessed on 27 April 2026). The implementation code and trained model weights are available upon reasonable request to the corresponding author.
